# The expression of Beta Human Chorionic Gonadotrophin (β-HCG) in human urothelial carcinoma

**Published:** 2010-12-16

**Authors:** Anthony Kodzo-Grey Venyo, David Herring, Harold Greenwood, Douglas John Lindsay Maloney

**Affiliations:** 1 North Manchester General Hospital, Department of Urology Delaunays Road Crumpsall Manchester M85RB, United Kingdom; 2 University Hospital of North Durham, Department of Surgery North Road Durham City DH1 5TW, United Kingdom; 3 University Hospital of North Durham Department of Pathology North Road Durham City DH1 5TW, United Kingdom; 4 Airedale General Hospital Department of Pathology Skipton Road Steeton Keigley West Yorkshire BD20 6TD, United Kingdom

**Keywords:** Urothelial, carcinoma, beta-HCG, Immunohistochemistry, Bladder Cancer

## Abstract

**Introduction:**

Studies regarding the immuno-histological expression and relevance of Beta-Human Chorionic Gonadotrophin (=-HCG) in urothelial carcinoma are few. There is also no clear cut way of predicting exactly which superficial urothelial carcinomas would subsequently recur or progress and which muscle-invasive urothelial tumours would progress. The objective of the study was to study the immunohistological expression of =-HCG in urothelial carcinoma with regards to grade, category and outcome following treatment

**Methods:**

The expression of =-HCG in urothelial carcinomas of 86 patients was studied with regards to grade, stage and outcome using an immunohistological (ABC) method and formalin fixed/paraffin embedded tumours.

**Results:**

Of the 86 tumours (55 superficial and 31 muscle-invasive) studied 45, 16 and 26 were graded as G1, G2, and G3 respectively. Thirteen of the 55 superficial tumours were positively stained for β=-HCG and 42 negatively stained. Twenty of the 31 muscle-invasive tumours studied were positively stained for β=-HCG and 11 were negative. Of the 13 β=-HCG positive superficial tumours only one did not recur at follow up and 12 subsequently recurred, of the 42 β=-HCG negative superficial tumours 19 did not recur and 23 recurred. Only one of twenty patients with β=-HCG positive muscle-invasive tumours survived; 6 of 11 patients with β=-HCG negative muscle-invasive tumours survived. The results indicate that positive staining of the tumours was more commonly associated with tumours of higher grade, higher stage and inferior outcome.

**Conclusion:**

The Immunohistological expression of β=-HCG would likely predict superficial tumours that would recur and muscle-invasive tumours with inferior outcome.

## Introduction

Currently there is no accurate way to predict which superficial urothelial cancers will subsequently become muscle invasive or which muscle invasive urothelial tumours will subsequently progress and result in death. Studies regarding the immunohistological expression of Beta Human Chorionic Gonadotrophin (β=-HCG) by urothelial cancers are few [1-4] and these studies have suggested varying rates of expression. This study was initiated to test the hypothesis that expression of β=-HCG in urothelial cancer is more commonly associated with tumours of high grade and high category and that the expression of β=-HCG is associated with inferior outcome.

## Methods

Between 1990 and 1994, 86 patients (49 male and 37 female), with urothelial carcinomata, mean age 69.5 years (range 20 to 95 years), treated in Dryburn Hospital were enrolled in the study. These patients had a mean follow up time of 55.7 months.

Urothelial tumour samples were obtained from all the 86 patients requiring surgical excision or transurethral resections of their tumours. 55 of these patients required transurethral resections of bladder tumours and 31 patients had resections of bladder tumours followed by radiotherapy and / or laparotomy and cystectomy. In each case the tumours were staged based upon the TNM classification (UICC 1987), by a careful bimanual examination under anaesthesia at the time of surgery in combination with the histology report. The tumours were graded according to the system of Bergkvist et al., using routine haematoxylin and eosin (H&E) stained sections of formalin fixed-paraffin embedded tumour. In addition, sections of 8-12 weeks gestational age placenta were obtained for use as positive control specimen for immunohistochemistry for β=-HCG. The patients were followed up at regular intervals and any recurrent or persistent tumour carefully graded and staged (categorised).

In the case of pTa and pT1 tumours, these patients had 3 monthly check cystoscopies initially for 2 years and in the absence of recurrence, check cystoscopies were carried out at 6 monthly intervals for 2 years following which, the patients were followed up at yearly intervals in the case of no recurrence but when a recurrent tumour was found the follow up interval was then reduced to 3 monthly intervals. Intravenous urography was performed at 2 yearly intervals and any recurrent or persistent tumour carefully graded and staged. The patients who had cystectomy were followed up in the out patients department (these patients had careful clinical examinations and appropriate investigations as was indicated for example bone scan chest X-ray, liver function test, intravenous urography, biopsy of any recurrent tumour as well as any other investigation and management that was necessary). Those patients who had transurethral resection of their tumours and subsequent radiotherapy were followed up by regular check cystoscopies and bimanual examination. In the case of the patients with superficial bladder tumours who had frequent superficial recurrences these patients were treated by intravesical chemotherapy following transurethral resection of their tumours.

**Figure 1:**
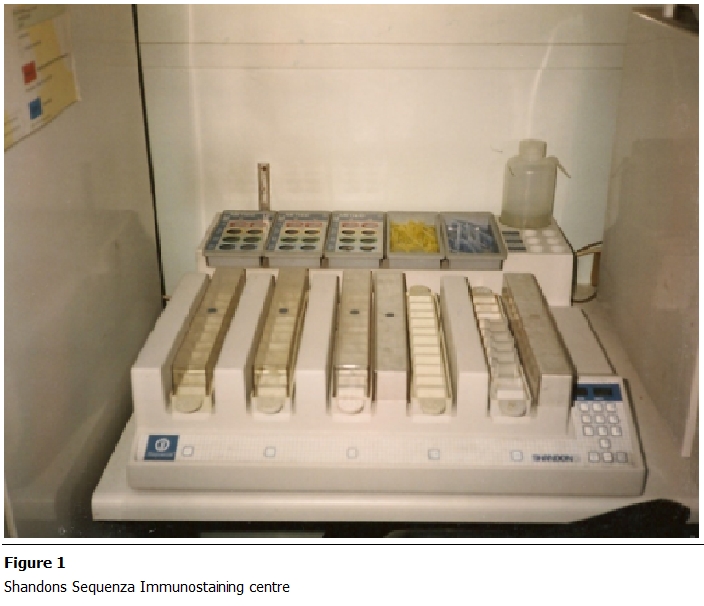
Shandons Sequenza Immunostaining centre

Routinely formalin fixed paraffin wax embedded blocks of urothelial cancer were cut at 5u and attached to poly-l-lysine coated slides. The sections were allowed to dry overnight at room temperature. The following Avidin-Biotin peroxidase (ABC) immunocytological procedure was then carried out: the sections were deparaffinised, rehydrated, rinsed in tap water for 5 minutes and then rinsed in distilled water. Endogenous peroxidase activity was blocked by placing the sections in 1% hydrogen peroxide/methanol solution for 20 minutes. The sections were next rinsed in running tap water for 5 minutes. In order to allow for “batch” runs to be performed and to provide consistent reproducible results, Shandons sequenza immuno-staining centre and its cover plate assembly (figure 1) was used for the subsequent intermediate steps of the immunohistochemistry. In fact the slides were put in the Shandons sequenza immunostaining centre and then rinsed in phosphate buffered saline (PBS) PH 7.2. Incubation was then carried out in normal rabbit serum (DAKO X902) diluted 1/20 with PBS for 10 minutes. The slides were next transferred to the primary antisera (β=-HCG antibody) diluted 1/100 with PBS (Sera Lab, monoclonal AB β=-HCG, AE8 319) and incubated overnight at 4 degrees centigrade. The slides were next rinsed in PBS for 5 minutes and then incubated in secondary antisera (DAKO E354 Rabbit anti-mouse immunoglobulins/Biotinylated) diluted with PBS for 5 minutes and then incubated in AB complex (DAKO K 355 AB Complex/HRP) diluted 1/50 with PBS for 45 minutes and rinsed in PBS for 5 minutes. Immunoreactivity was visualised with Diaminobenzidine Tetrachloride dihydrate (DAB) Page number not for citation purposes 3 solution for 1 to 5 minutes. The slides were rinsed in PBS for 5 minutes and then removed from the sequenza immunostaining centre and rinsed in running tap water for 10 minutes. The cell nuclei were counter stained lightly in Mayers Haematoxylin. The slides were washed, dehydrated, cleared and mounted in DPX.

Sections of formalin fixed paraffin wax embedded placenta were also cut at 5u and stained simultaneously with the urothelial tissues using the same steps as above for use as control slides. For purposes of negative control, sections of tumour specimens were processed and stained as above apart from the omission of the primary antisera.

### Microscopy (immunohistochemistry for β-HCG)

**Figure 2:**
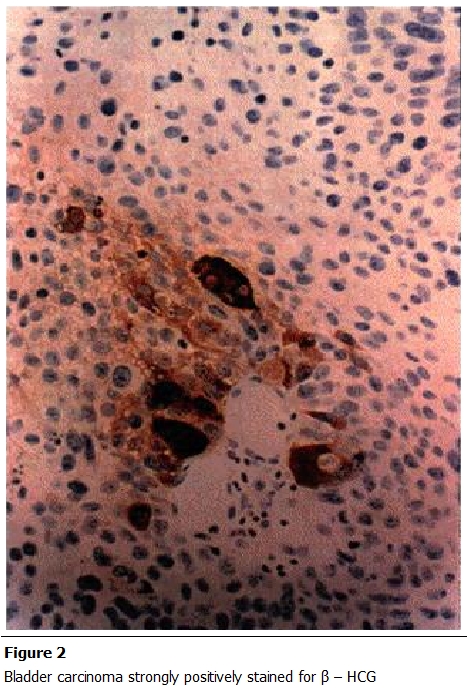
Bladder carcinoma strongly positively stained for β – HCG

Routine microscopy of the immunohistochemistry slides was performed in order to assess each slide for the expression of β=-HCG (staining for β=- HCG). Positive staining was demonstrated by brownish coloration in the cytoplasm of tumour cells similar to the staining characteristics of the placenta (Figure 2).

### Assessment of staining for β-HCG

Staining was assessed taking into consideration the intensity of positive staining throughout the section. Staining intensity was scored on a 4 point scale: Negative stain (No staining) (0), weak (1), moderate (2), and strong (3). The extent of staining was based upon the proportion of tumour cells positively stained: 0-25% (+), 26-50% (++), 51-75% (+++), 76 -100% (++++). The extent of staining was summarised as: a - for + and ++ and b for +++ and ++++. In the final analysis of data all tumours showing weak, moderate and strong staining were recorded as positive and those showing no staining (0) were recorded as negative. Tumour cytoplasmic staining was recorded as positive.

### Statistical analysis

Statistical analysis of the results was done using SPSS for windows to calculate chi square tests of the various tumour groups and the outcome.

## Results

The results of the histological and immunohistochemical analysis of the tumours are summarised in Table 1 and 2. Forty five tumours were well differentiated (G1), 15 tumours were moderately differentiated (G2) and 26 tumours were poorly differentiated (G3). Thirty one tumours were muscle invasive tumours and the remaining 55 tumours were pTa and pT1 tumours. Positive staining of any intensity (mild, moderate and strong staining) irrespective of the proportion of tumours stained was obtained in 33 of the 86 tumours. Eleven of the 45 well differentiated (G1) tumours (24%) were positively stained for β=-HCG and 5 of the 15 moderately differentiated (G2) tumours (33%) were positively stained for β=-HCG and 17 of the 26 poorly differentiated (G3) tumours (65%) were positively stained for β=-HCG. Expression of β=- HCG was found to be more common in higher grade tumours. Analysis of the data using chi square test confirmed that there is a significant difference in the staining characteristics of the three grades of tumour (p < 0.01). Thirteen of the 55 pTa and pT1 (superficial) tumours (24%) had positive staining of any intensity (mild, moderate and strong staining) for β=-HCG and 20 of the 31 muscle invasive (T2 - T4) tumours (66%) had positive staining of any intensity (mild, moderate or strong staining) for β=- HCG. A correlation between positive staining for β=-HCG and higher tumour category was apparent. The difference in the staining characteristics of the superficial and muscle-invasive tumour groups was found to be significant using chi-square test (p < 0.01).

The results of the staining characteristics of the tumours and outcome are illustrated in Table 3 and 4. Only 1 of the 20 pTa and pT1 (superficial) tumours (5%) which did not recur was β=- HCG positive the remaining 19 were negatively stained for β=- HCG. Twelve of the 35 tumours (34%) that Page number not for citation purposes 4 recurred were positively stained for β=- HCG. Regarding the 21 tumours in which the recurrences were of the same grade and tumour category, 6 were β=- HCG positive (29%). Of the 14 tumours in which the recurrences were of higher grade and / or higher category, 6 (43%) were positive for β=- HCG. A correlation between negative staining and no recurrence of tumour at follow up was observed. Superficial tumours that recurred on analysis were found to have a higher proportion of β=- HCG positive tumours in comparison with tumours in which no recurrences were observed. (p < 0.05). It was also observed that positive staining for β=- HCG was associated with recurrence of higher grade and higher stage (category). Only 1 of the 20 patients with β=-HCG positive muscle invasive tumours (5%) survived following treatment (radiotherapy and or cystectomy). In comparison, 6 patients out of the 11 (55%) with tumours negative for β=- HCG were alive. The difference in the outcome of the muscle invasive tumours based upon their staining characteristics was found to be significant (p < 0.01).

## Discussion

The results of this prospective study would confirm the hypothesis that expression of β=-HCG is common in tumours of higher grade and higher category as well as is associated with inferior outcome. Studies regarding the immuno-histological expression of β=-HCG by urothelial cancers are few, and these studies have suggested varying rates of expression. Rodenburg et al. [1] found 5 of 13 transitional cell carcinoma cases (38.5%) stained positive for β=-HCG by using immunoperoxidase technique. Shah et al. [2] studied the expression of β=- HCG in 104 bladder cancer cases of various stages and grade and found 12 positive (11.5%), all were poorly differentiated. Shah et al. [3] also studied the expression of β=-HCG of 14 upper tract tumours by immuno-histochemistry and found 2 positive (14.3%). Tungekar et al. [4] studied 22 cases of schistosomiasis associated squamous cell carcinoma of bladder for β=-HCG expression by immuno-histochemistry and found 2 moderately differentiated and 2 poorly differentiated T2 to T4 tumours were positive (18%). On the other hand, Martin et al. [5] studied 100 cases of invasive (T2/T3) transitional cell carcinoma of bladder for the expression of β=-HCG by immuno-histochemistry and found 29 (29%) were positive. They also found that the expression of β=-HCG correlated with tumours that did not respond to radiotherapy and the presence of squamous metaplasia. In a study of malignancies producing ectopic HCG, which included 2 bladder cancers, Crawford et al [6] suggested that such expression may indicate chemosensitivity. On the contrary it has been suggested that tumours expressing β=- HCG may be radio-resistant [7-10].

The results of this study have revealed a significant proportion of transitional (urothelial) carcinomas to be associated with the expression of β=- HCG, 33 of the 86 (38%) were positive for β=-HCG. The proportion of T2 to T4 tumours that were β=- HCG positive in this study is high. Twenty out of 31 tumours (65%) were β=-HCG positive. Superficial tumours of higher grade tend to be associated with more recurrences in comparison with low grade tumours. In this study there was only 1 G3 superficial tumour therefore it was not possible to find out whether the observed high incidence of recurrence associated with expression of β=-HCG is independent of grade. The observation that 19 out of 20 patients with β=-HCG positive T2 -T4 tumours died within 4 years may be important. The significance of this observation is not known due to the fact that the number of patients involved is small. Other factors including nodal involvement of a muscle-invasive tumour may have an adverse effect on outcome. However a number of muscle-invasive tumours without nodal involvement were associated with poor outcome. The latter observation may suggest the possibility that radiotherapy alone or cystectomy alone may not be adequate treatment for such tumours. An explanation for such a grave prognosis may be the fact that it has been suggested that the expression of β=-HCG is associated with either defective or blocked cellular immune capabilities of the patients.[11] Depression of cell-mediated immunity associated with the expression of β=-HCG may be either generalised or specific, the latter being a form of immune tolerance. On the other hand, generalised depression of cellular immunity in patients with tumours expressing β=-HCG may perhaps be characterised by an impaired or absent response of blood lymphocytes to the tumours.

Although it was observed that the expression of β=-HCG was associated with inferior prognosis, there were a few β=- HCG negative tumours that progressed resulting in the death of the patients. It would therefore be worthwhile to find out whether or not there is another tumour marker which would pick out tumours with inferior prognosis which had not been identified by immuno-histochemistry for β=-HCG. Considering the fact that the nodal status of the muscle-invasive tumours was not taken into consideration in this study there is the need to conduct another study Page number not for citation purposes 5 recruiting a large number of patients to investigate whether or not the inferior prognosis associated with the expression of β=HCG is independent of the nodal status of the muscle-invasive urothelial carcinomas.

Dirnhofer and associates [12] designed an immunohistochemical study to assess the trophoblastic hormone production profile of transitional cell carcinoma of the bladder. They correlated histological differentiation and tumour stages with marker expression as well as evaluated a potential tumour origin of hCGbeta core-fragment (hCGbetacf). In this study formalin-fixed, paraffin-embedded tumour tissues from 104 patients with urothelial neoplasms of various histological grades (23 G1, 24G2, and 38 G3) and stage (19pTis, 21pTa, 29pT1, and 35 pT2-T4) were analyzed by the immunoperoxidase technique using their own well-characterized monoclonal antibodies against the glycoprotein hormones human chorionic gonadotropin (hCG) and its derivatives hCGalpha, hCGbeta, hCGbetacf, luteinizing hormone (LH), LHbeta), follicle-stimulating-stimulating hormone (FSH, FSHbeta), and the protein hormones placental lactogen (hPL) and growth hormone ((hGH-V/N). Dirnhofer and associates [12] found that: Overall, trophoblastic hormone immunoreactivity in 36% of transitional cell carcinoma, and detailed analysis showed 35 % hCGbeta, 17% hCGbetacf, 9% hCGalpha, 4% HCG, and 2% hPL-positive cases. The tumours produced neither GH-N, placental GH-V, nor the pituitary gonadotropins FSH/FSHbeta and LH/LHbeta. Marker positivity significantly increased with high-grade lesions (26% G1 v 55% G3 transitional cell carcinomas) and advanced tumour stages (24% pTa v 63% > or = pT2). Hormone immunoreactivity was frequently observed in highly proliferating areas. Normal urothelium, urothelial papillomas, and carcinoma in situ showed no positive reactions. All tumours producing hCGderived molecules were negative for the concomitantly analyzed neuroendocrine markers chromogranin A, synaptophysin and neuron-specific enolase (NSE). Dirnhofer and associates [12] were of the opinion that their findings summarized above, together with recent structural and clinical studies strongly suggest that these hormones, or derivatives thereof, might act as local growth factors.

The beta-subunit of human chorionic gonadotrophin (hCG) is coded on chromosome 19 by the beta-hCG-hLH gene cluster. Iles and associates [13] isolated Genomic DNA from bladder tumour cell lines which ectopically express β=-HCG. The beta-hCG-hLH gene cluster was probed for possible rearrangement or amplification and cells karyotyped for chromosome 19 abnormalities. They did not find any rearrangement or amplification of the gene cluster and no consistent abnormalities of chromosome 19 were found. Iles and associates [13] concluded that the expression of beta-hCG by bladder tumours is therefore likely to be the result of altered gene regulation and not a rearrangement or amplification of this gene cluster.

It has been suggested that ectopic production of free hCG beta is a common phenomenon in epithelial tumours, a phenomenon originally believed to have no biological significance [14]. However, it is now apparent that hCG beta may significantly affect tumour development by increasing cell populations through inhibition of apoptosis [14]. The hCG beta beta homodimer, with topological similarities to cystine knot growth factors, has been suggested to be the responsible mediator of these novel tumourigenic responses [14]. Butler and Iles [14] isolated hCG beta monomer from hCG beta beta homodimer using sixe exclusion chromatography and confirmed the separation by Western blotting. Using a tetrazolium bromide incorporation cell number quantification assay (MTT), they measured the growth effects of separated hCG beta fractions corresponding to monomeric (hCG beta) and dimeric (hCG beta beta) forms on the hCG beta responding cell line T24. They observed that maximal increases in cell number corresponded to the elution peak of dimeric and monomeric hCG. Butler and Iles [14] concluded that: It would appear that the recently observed hCG beta beta homodimer is no more bioactive than its monomeric counterpart, in stimulating bladder cancer cell growth. This strengthens the proposition that hCG may exert its antiapoptotic effects by antagonistic inhibition of other cystine knot growth factor receptors and not by a specific receptor-mediated homodimer interaction as seen for its topological counterparts TGF, PDGF-B and NGF.

Butler and associates [15] proposed that the ectopic production of hCG beta was contributing in an autocrine fashion to the radio-resistance and metastatic potential of such secreting tumours. They also demonstrated that the addition of hCG beta to the culture media of bladder, cervical and endometrial carcinoma cells lines brought about an increase in the rate of replication. Since a cell population size is a balance of mitosis and mortality, Butler and associates [15] proposed that hCG beta was inhibiting apoptosis. Butler and associates [15] demonstrated that following incubation with recombinant hCG beta, bladder carcinoma cells refrain from undergoing apoptosis. Butler and associates [15] carried out quantification of apoptosis bodies by radioimmunoassay and corrected to cell number as determined by MTT assay. In each cell line, addition of hCG beta reduced the number of apoptotic bodies dose-dependently, indicating a diminished apoptotic rate. Butler and associates [15] suggested that furthermore, TGFbeta1-induced apoptosis could be dose-dependently inhibited by co-incubation with hCG beta. Butler and associates [15] Page number not for citation purposes 6 therefore proposed that: Such a decline in apoptosis may account for the cell population increase previously reported. It may also explain the radio-resistance and aggressive nature of hCG beta-secreting tumours and the poor prognosis associated therein.

The effects of human chorionic gonadotrophin (hCG) and its subunits on in vitro bladder cancer cell growth assessed by Gillot and associates [16] using the tetrazolium salt reduction assay (MTT), intact hCG, alpha-hCG and beta-core hCG were all found to have no effect on cell growth, while beta-hCG increased MTT reduction in all bladder cell lines tested. The magnitude of beta-hCG stimulation were observed to be maximal in the T24 line, which does not itself produce beta-hCG and appeared to be correspondingly lower in beta-hCG-secreting lines. The addition of antibodies to beta-hCG inhibited MTT reduction among high secretors but failed to inhibit MTT reduction in non-beta-hCG producers. Gillot and associates concluded that the results are consistent with the poor prognosis associated with beta-hCG expression by bladder tumours in vivo and suggest an autocrine/paracrine stimulation of tumour growth by endogenously produced beta-hCG.

Iles and Chard [17] stated that material with immunochemical characteristics of human chorionic gonadotrophin (hCG) is produced by bladder tumour cells in vitro and in vivo. In order to characterize this material further, Iles and Chard [17] collected media from 17 cell cultures (three choriocarcinomas, seven bladder carcinomas and seven “normal” urothelium). The hCG-like material was compared with pregnancy hCG and purified alpha- and beta-subunits by specific radioimmunoassay by SDS-PAGE and Western blotting. It was shown that both the neoplastic and normal urothelium produced only free beta-subunit-like material. Iles and Butler [17] concluded that this urothelial “beta-hCG” has the same molecular weight and electrophoretic mobility as that present in the intact hCG of pregnancy.

Iles and associates [18] measured human chorionic gonadotrophin (hCG) and alpha-feto-protein (AFP) in culture media from a panel of 29 cell lines including 9 bladder carcinomas, 5 'normal' bladder epithelia, 10 germ cell tumours, and 5 miscellaneous tumours and 'normal' cell lines. In 7 of the 9 bladder carcinomas and 4 of the 5 'normal' bladder epithelia, the media contained hCG at levels ranging from between 34 and 3,600 IU 1(-1). All other cell lines, including the 10 germ cell tumour lines yielded negative results for hCG. Iles and associates [18] concluded that these findings indicate that in vitro secretion of hCG is a common feature of normal and neoplastic bladder transitional epithelia, and support the hypothesis that parts of the genitor-urinary epithelium have a potential for hCG production.

Moutzouris and associates [19] studied the biopsies from 75 patients with transitional cell carcinoma of the bladder (25 Ta-T1, 45 T2-T4, 5M) immunohistochemically for the expression of beta-human chorionic gonadotrophin (beta-HCG). They found that: 1) Only 5 Ta – T1 tumours contained a small number of beta-HCG positive cells but 24 invasive tumours and all patients with metastases showed increased numbers of positive cells; 2) A significant correlation existed between beta-HCG immunoreactivity and tumour category.

In 30 patients with muscle-invasive disease (T2-T4, N0, M0) who were treated with radical radiotherapy, a significant correlation existed between response to treatment and beta-HCG expression; beta-HCG positive tumours did not respond to treatment. A difference in survival was found between patients with tumours negative for beta-HCG compared with patients with positive tumours, all treated with radical radiotherapy.

They concluded that the results indicate that beta-HCG expression increased with tumour invasiveness and the use of immunohistochemistry may prove a useful means of identifying radioresistance and aggressive forms of bladder cancer.

Iles and Chard [20] showed that interferon-alpha (IFN-alpha) enhances the ectopic production of the beta-subunit of hCG by bladder tumour cells. Iles and Chard also stated that their study suggested a direct transcription/translational effect of this cytokine (IFN-alpha) on the expression of a reproductive endocrine gene.

It has been stated that human chorionic gonadotropin (hCG) is a marker of trphoblasric tumours and the serum concentration of the free betasubunit is an independent prognostic marker in several non-trophoblastic cancers [21]. hCGbeta is encoded by six genes of which the type II genes (hCGbeta 3/9, 5 and 8) are thought to be upregulated in relation to type I genes (hCGbeta 6/7) in cancer [21]. Hotakainen and associates Page number not for citation purposes 7 [21] developed a novel quantitative RT-PCR method for the quantification of the relative expression levels of the two groups of hCGbeta genes and analyzed 28 bladder tumours and 15 urine samples. They found that a higher relative expression level of type II genes in malignant compared with benign urothelia (p = 0.016) and in exfoliated urinary cells from cancer patients compared with those from benign controls (p = 0.026). The expression level was increasing with higher stage (p = 0.014) and grade (p = 0.001) and tended to be higher in relapsing tumours (p = 0.059). Hotakeinen and associates [21] concluded that the increased hCGbeta concentrations in body fluids of patients with aggressive bladder cancer may be due to over-expression of typeII genes. They recommended that Quantification of the relative mRNA expression levels of the hCGbeta type I and II genes in urine cells should be further studied as a potential non-invasive tool for the diagnosis and follow-up of bladder cancer.

Iles and associates [22], measured Beta human chorionic gonadotrophin (beta HCG)in 127 urine and 85 serum samples from 175 untreated patients with urothelial cancer. They found that serum levels of beta HCG were substantially elevated in 16 of 21 patients (76%) with widespread metastases but in only 2 of 64 patients (3%) with disease confined to the pelvis. Urine beta HCG levels were moderately raised in 11 of 25 patients (44%) with locally advanced disease but greatly elevated in 5 of 7 patients (71%) with metastases. Iles and associates [22] concluded that measurement of serum and/or urine beta HCG appears to be an efficient diagnostic marker for the presence of distant metastases in bladder carcinoma.

Dobrowolski and associates [23] assessed Beta human chorionic gonadotrophin levels in blood serum of 79 patients with bladder tumours before and seven days after trans-urethral resection of the tumours. They found that the mean serum beta HCG levels increased with the growth grade of anaplasia and staging. They concluded that Beta HCG was a good biological marker to differentiate between superficial and deep tumours.

Smith and associates [24] assessed the potential value of ectopic beta-human chorionic gonadotrophin (beta HCG) measurement in the clinical management of transitional cell carcinoma (TCC). They conducted a prospective serological study of 163 consecutive patients undergoing cystoscopy as new or review cases to assess any correlation between beta HCG production and histological grading or stage. They found that ten per cent of patients with TCC had raised beta HCG level but there was no correlation with tumour differentiation, staging or prognosis. They concluded that beta HCG has no role as a tumour marker for TCC and therefore appears unlikely to play a part in the clinical management or treatment of urothelial tumours.

Mora and associates [25] evaluated the clinical performance of assays measuring intact human chorionic gonadotropin alone (i-hCG), intact and nicked human chorionic gonadotropin (i-hCG and hCGn), free beta-subunit (free beta-hCG) and total beta-human chorionic gonadotropin (t-hCG) using different commercial kits, in a group of bladder carcinoma patients with ectopic human chorionic gonadotropin (hCG) secretion, at diagnosis and during treatment. The diagnostic sensitivity obtained ranged between 63.6% and 75.7% (t-hCG assays), 72.7% (free beta-hCG assay), 18.2% (i-hCG and hCGn) and 6% (i-hCG assay). Median increases of hCG during treatment in patients with chemotherapy resistance ranged from 4.9 to 6.9 for t-hCG and free beta-hCG assays and from 1.4 to 3.2 for i-hCG and i-hCG plus hCGn assays. Observed median decreases when chemotherapy was efficient ranged from 2.8 to 3.3 (t-hCG and free beta-hCG assays) and from 1.1 to 1.5 (i-hCG and i-hCG plus hCGn assays). They concluded that t-hCG and free beta-hCG are the most suitable assays for the management of bladder carcinoma patients as the ectopic secretion of chorionic gonadotropin is mainly due to the free beta-subunit.

Iles and associates [26] examined the prognostic significance of elevated urinary beta human chorionic gonadotrophin (beta-hCG) in patients with bladder cancer. They measured total beta-hCG in the urine of 142 patients referred for cystoscopic examination. The patients were followed up for a minimum of 17 months and grouped according to stage of disease. In view of the fact that the water output by individual patients varied, urinary creatinine levels were measured as an indicator of the concentration of the urine sample. Patient outcomes were correlated with urinary total betahCG levels both corrected and uncorrected for ceatinine concentration. After correcting the creatinine levels, 40 patients were excluded because the sample was too dilute; (undetectable beta-hCG and a creatinine level of < 4 mmol/L). A further four patients were excluded because they had concurrent malignancies not in the urinary bladder and one patient was lost to follow-up. Iles and associates [26] found that none of the 52 patients with benign conditions, nine of the 27 with Ta-T1, and nine of the 25 with T2-T4 bladder disease had urinary beta-hCG levels > 3.74 IU/L creatinine. There was no significant association between urinary total beta-hCG concentration and the rates of recurrence or progression for Ta – Page number not for citation purposes 8 T1 disease at 17 months of follow-up. In the case of patients with T2-T4 disease there was significant association with widespread metastasis (P < 0.01) and mortality, P = 0.07; Kaplan-Meier survival time analysis uncorrected for creatinine P = 0.027, corrected for creatinine P < 0.001). This association could not be accounted for by differences in age, histopathology or treatment. Iles and associates [26] concluded that although sample concentration was a serious confounding factor, after correcting for dilution using creatinine content, the elevated urinary levels of total beta-hCG indicated those T2-T4 lesions which were likely to metastasize and those patients likely to die early. They stated that if this test is to be used clinically, concentrated samples, i.e. early-morning urine, and a more sensitive beta-hCG assay are required. However, for T2-T4 bladder tumours, an elevated pre-treatment level of urinary beta-hCG is a marker of poor prognosis and may prove useful in deciding appropriate therapy.

Venyo and associates [27] undertook a study in order to study the levels of =-HCG in sera of patients with urothelial carcinomas with regards to the association with histological grade, tumour category and outcome in order to achieve the following aims: 1) to find out whether or not raised serum levels of =-HCG are associated with higher grade and higher category tumours, 2)to determine whether in patients with raised levels of =- HCG in their sera the rise (above normal range) and the fall (to normal) in =-HCG levels would correspond with presence and absence of tumour respectively hence serum levels could be used as an additional way of monitoring the progress of urothelial tumours, 3) to investigate the hypothesis that elevation of serum levels of =-HCG in urothelial carcinomas is associated with inferior prognosis, 4)to determine whether or not serial measurements of serum levels of =-HCG could be used to monitor the progress of urothelial cancer.

Venyo and associates [27] performed radioimmunoassay of sera (blood samples) of 120 patients (mean age 70 years; range 25 - 95 years) with urothelial carcinomas at the time of tumour diagnosis and at follow-up. For control purposes radioimmunoassay of sera were performed in two groups of patients: Group A: 30 patients with benign conditions who came for operations like hernia repair; Group B: 70 patients who previously had resection of superficial urothelial who repeatedly had no evidence of recurrent tumours at review cystoscopy. Venyo and associates [27] found that all the 30 patients in the control group A had normal levels of serum β=-HCG (< 4 IU/L); 1 patient out of 70 patients in Group B who had no evidence of recurrence at review cystoscopy described above had a slightly elevated level of β=-HCG (this patient had a red patch but no tumour found at histology). Statistical analysis using Mann Whitney test and Box plot showed that the control group A and group B were the same or similar. (Mann Whitney U (control group A v no recurrence group B); U = 1009; Z = 0.090 p = 0.756). 36 (30%) of the 120 patients in the tumour group had raised levels of serum =-HCG ranging from 5 to 12241 IU/L. Patients with metastatic disease had higher levels than those with nonmetastatic disease. Statistical analysis using Mann Whitney test and Box plot showed a significant difference between the tumour group and the control group A as well as a significant difference between the tumour group and the no recurrence group B (Mann Whitney U (control group A v tumour group U = 893 Z = -4.2803 p < 0.001; no recurrence group B v tumour group U = 2228 Z = -5.4088 p = < 0.001). Venyo and associates [27] summarized their findings as follows: 1) 30% of all the urothelial carcinomas of all grades and stage were associated with raised serum levels of β=-HCG, 2) Grades 1, 2 and 3 tumours were associated with raised levels of serum β=-HCG in about 19%, 39%, and 47% of the tumours respectively and hence the higher the histological grade the higher the proportion of tumours associated with raised levels of serum β=-HCG, 3)About 23% of the superficial tumours were associated with raised levels of serum β=-HCG compared with about 47% of muscle-invasive urothelial tumours. Muscle-invasive tumours therefore tend to be associated with raised levels of serum β=-HCG compared with superficial tumours.

Muscle-invasive tumours associated with raised levels of serum β=-HCG had inferior prognosis after radiotherapy and or cystectomy in comparison with muscle-invasive tumours associated with normal levels of serum β=-HCG treated by radiotherapy or cystectomy.

Venyo and associates [27] concluded that: 1) The serial measurement of serum =-HCG may prove to be a useful adjunct to the follow-up of patients whose tumours are associated with raised levels of β=-HCG in their blood provided the elevation of the serum β=-HCG level is due to production by the tumour, 2) The measurement of serum β=-HCG in urothelial carcinoma may prove to be a method of identifying muscle-invasive tumours (T2-T4) which should be treated aggressively, perhaps, by adjuvant systemic chemotherapy, 3) the presence of β=-HCG in urothelial tumours may add some prognostic information to the heterogenous biological behaviour of such tumours (usually aggressive tumours). Lazar and associates [28] stated that in view of the fact that increased serum levels of human chorionic gonadotrophin beta subunit (hCG beta) had previously been described in patients with bladder cancer they undertook a study to obtain insight into such production of hCG beta. They Page number not for citation purposes 9 investigated the expression of hCG beta 7, 8, 5, and 3 genes in bladder carcinomas and normal urothelia by reverse transcription PCR. They detected surprisingly, hCG beta mRNAs in both normal urothelia and carcinomatous cells

Nevertheless, tumour progression was characterized by different patterns of transcription of the hCG beta genes; the beta 7 gene was the only gene transcribed in normal urothelia and Ta tumours included in the study, whereas in addition to beta 7, genes beta 5, 8, and 3 were transcribed in T1 to T4 tumours. In addition, transcription levels of the latter three genes increased with the stage of the disease. Lazar and associates [28] concluded that these observations showed that dramatic modifications in the expression of hCG beta genes accompany progression of bladder carcinomas.

Geissler and associates [29] attempted to generate cytotoxic T lymphocytes (CTL) with activity against free hCG beta-producing tumours by genetic immunization using a construct containing a beta subunit expressing cDNA. In order to assess CTL activity in vivo, a cloned syngeneic SP2/0 myeloma cell line was established that constitutively expresses the free hCG beta protein. Inoculation of this cell line into BALB/c mice produced large tumours within 2 weeks. Nevertheless, mice immunized with the free hCG beta expression construct demonstrated a marked reduction of tumour size and weight compared with animals immunized with mock DNA (“empty” plasmid). It was found that 30% of immunized mice were tumour-free after 3 months and thus considered long-term survivors. Inhibition of tumour growth was strongly associated with the level of CTL activity present in CD8+ cells derived from the spleen. Also, immunized mice developed high titer anti-hCG beta antibodies that neutralized the biologic effects of the intact hCG glycoprotein hormone on its cellular receptor as well. Geissler and associates [29] concluded that: these results illustrate that substantial cellular and humoral immune responses to the free hCG beta subunit may be generated by DNA immunization; this study presents a potential approach to inhibiting growth of human tumour cells that produce and secrete the free hCG beta protein.

He and associates [30] described the development of a novel antibody-based dendritic cell (DC)-targeted cancer vaccine capable of eliciting cellular immune responses directed against hCGbeta. They coupled the tumour-associated antigen hCG genetically to a human anti-DC antibody (B11). The resulting fusion protein (B11-hCGbeta) was evaluated for its ability to promote tumour antigen-specific cellular immune responses in a human in vitro model. Monocyte-derived human DCs from normal donors were exposed to purified B11-hCGbeta, activated with CD40 ligand, mixed with autologous lymphocytes, and tested for their ability to promote hCGbeta-specific proliferative and cytotoxic T-lymphocyte responses.

The following results were obtained: 1) B11-hCGbeta was found to be a soluble, well-defined and readily purified product that specifically recognized the human mannose receptor via the B11 antibody portion of the fusion protein, 2) B11-hCGbeta functionally promoted the uptake and processing of tumour antigen by DCs, which led to the generation of tumour-specific HLA class I and class II-restricted T-cell responses, including CTLs capable of killing human cancer cell lines expressing hCGbeta.

He and associates [30] concluded that: although other hCG vaccines have been shown to be capable of eliciting antibody responses for hCGbeta, this is the first time that cellular immune responses to hCGbeta have been induced by a vaccine in a human system; this DC-targeted hCGbeta vaccine holds promise for the management of a number of cancers and merits additional clinical development.

## Conclusion

The results of the study indicate that: 1) Superficial tumours that are positively stained for β=-HCG are more likely to be associated with future recurrence in comparison with tumours that are negatively stained for β=-HCG , 2) Muscle-invasive urothelial tumours that are positively stained for β=-HCG have inferior prognosis in comparison with muscle-invasive tumours that are negatively stained for β=-HCG.

## Ethical Approval

Ethical approval for this project was obtained from the Durham Health Ethical Committee (North Durham Ethics Committee).

## Competing interests

This paper was presented at the Bi-Annual meeting of the South African Urological Association in Pretoria South Africa 1994 and at the Bi-Annual Meeting of the Pan African Urological Surgeons Association in Nairobi Kenya in 1995. We do not have any conflict of interest to declare.

## Authors' Contribution

Anthony Kodzo-Grey Venyo designed the study, applied for and obtained ethical approval for the project, recruited patients for the study, was involved in the following: assessment, investigation, management and follow-up of the patients including clinical staging of all the tumours. He also collected all urothelial tumour specimens for storage, cutting and staining. He studied the technique of cutting and staining of specimens under Mr Harry Greenwood. He also undertook microscopic examination of all immuno-histologically stained slides for scoring of the staining characteristics of the tumours and the control specimen. Additional tasks performed by Anthony Kodzo-Grey Venyo include: Microscopic examination of all the tumour specimens that had already been graded and staged by Dr Douglas John Lindsay Maloney; He analyzed the results of all the histological data and correlated them with the clinical data; the entire write up of the paper. David Herring agreed to be a co-supervisor for the project. He was involved with the assessment, investigation and management of the patients from the beginning to the end of the project and he read all the report and agreed with all the findings. Harry Greenwood collected all the tumour specimens from Anthony Kodzo-Grey Venyo, stored and proceesed all the specimens for the routine haematoxylin and eosin staining and wells as the immuno-histological staining of all the specimens. (He stained / supervised all the staining). He also examined and cross checked the immuno-histological findings of Anthony Kodzo-Grey Venyo. Mr Greenwood also read the paper which has summarized our findings. Douglas John Lindsay Maloney agreed to be a joint supervisor for the project. Dr Maloney did the histological grading and pathological staging of all the tumours and he also checked the immuno-histologically stained specimens for their staining characteristics as well as he read the paper which has summarized our findings. All the authors have read and approved the final version of the manuscript.

## Acknowledgements

Mr. PJ English (formerly Consultant Urologist at Dryburn Hospital Durham City United Kingdom and now Consultant Urologist at Sunderland Royal Infirmary United Kingdom) for his support; and Professor David E. Neal (formerly Professor of Surgery in University of Newcastle-upon Tyne Medical School United Kingdom and currently Professor and Chairman of Oncology at Cambridge in the United Kingdom) for his supervising guidance, support and help throughout the research project and its write up. We are also grateful to Dr Thompson Sarkordie-Djan former Senior Lecturer in Engineering at Tees Side University in Middlesbrough in the United Kingdom for his help with the statistical analysis.

## Figures and Tables

**Table 1:** Expression of β-HCG and histological grade of urothelial carcinomas (86 patients - Formalin fixed paraffin embedded tissue)

	**GRADE**			
	**G1**	**G2**	**G3**	**Total**
Positive	11	5	17	33
Negative	34	10	9	53
Totals	45	15	26	86

**Table 2:** Expression of =-HCG and tumour category of urothelial carcinomas (86 patients - Formalin fixed paraffin embedded tissue)

	Cis+pTa+pT1 tumours*	**T2-T4 tumours^$^**	**Totals**
Positive	13	20	33
Negative	42	11	53
Totals	55	31	86

*Superficial tumours (Cis+pTa+pT1) ; $ muscle-invasive (T2-T4) tumours

**Table 3:** Expression of β - HCG and outcome of pTa and pT1 tumours

Outcome	**β – HCG positive**	**β – HCG negative**	**Totals**
No Recurrence	1	19	20
Recurrence of same grade & category	6	15	21
Recurrence of higher grade & same category	4	4	8
Recurrence of higher stage (category)	2	4	6
Totals	13	42	55

**Table 4:** Expression of β=- HCG and outcome of Muscle Invasive (T2 -T4) tumour Group. (31 patients, treated by radiotherapy and or laparotomy +/- cystectomy)

**Outcome**	**B – HCG positive**	**B – HCG negative**	**Totals**
Alive	1	6	7
Died as a result of tumour	19	5	24
Totals	20	11	31

## References

[1] Rodenburg C J, Nieuwenhuyzen Kiruseman A C, de maaker H A, Fleuren G J, Van Oosterom A T (1985). Immunohistochemical localisation and chromatographic characterisation of human chorionic gonadotrophin in bladder carcinoma.. Arch Pathol Lab Med..

[2] Shah V M, Newman J, Crocker J, Chapple C R Collard, M J, O'brien J M, Considine J (1986). Ectopic beta-human chorionic gonadotropin production by bladder urothelial neoplasia.. Arch Path Lab Med..

[3] Shah V M, Newman J, Crocker J, Antonakopoulos G N, Chapple C R, Collard M J (1987). Production of beta-human chorionic gonadotropin by prostatic adenocarcinoma and transitional cell carcinoma of the upper urinary tract.. Brit J Exp Path..

[4] Tungekar M F, Abdul-Sattar S, Adnani M S (1988). Expression of chorionic gonadotropin by schistosomiasis associated squamous cell carcinomas of bladder.. Eur Urol..

[5] Martin J E, Jenkins B J, Zuk R J, Oliver R T D, Baithun S I (1989). Human chorionic gonadotrophin expression and histological findings as predictors of response to radiotherapy in carcinoma of the urinary bladder.. Virchows Arch A Pathol Anat Histopathol..

[6] Crawford S M, Ledermann J A, Turkie W, Rustin G J S, Begent R H J, Newlands E S, Bagshawe K D (1986). Is ectopic production of human chorionic gonadotrophin (HCG) or alphafetoprotein (AFP) by tumours a marker of chemosensitivity?.. Eur J Cancer Clin Oncol..

[7] Obe J A, Rosen N, Koss L G (1983). Primary choriocarcinoma of the urinary bladder Report of a case with probable epithelial origin.. Cancer..

[8] Burry A F, Munn S R, Arnold E P, Mc Rae C U (1986). Trophoblastic metaplasia in urothelial carcinoma of the bladder.. Br J Urol..

[9] Dennis P M, Turner A G (1984). Primary choriocarcinoma of the bladder evolving from transitional cell carcinoma.. J Clin Path..

[10] Ainsworth R W, Gresham G A (1960). Primary choriocarcinoma of the urinary bladder in a male.. J Path Bact..

[11] Adcock E W 111, Teasdale F, August C S, Cox S, Meschia G, Battaglia F C, Naughton M A (1973). Human chorionic gonadotropin its possible role in maternal lymphocyte suppression.. Science..

[12] Dirnhofer S, Koessler P, Ensinger C, Feichtinger H, Madersbacher S, Berger P (1998). Production of trophoblastic hormones by transitional cell carcinoma of the bladder: association to tumor stage and grade.. Hum Pathol..

[13] Iles R K, Czepulkowski B H, Young B D, Chard T (1989). Amplification or rearrangement of the beta-human chorionic gonadotrophin (betahCG)—human LH gene cluster is not responsible for the ectopic production of beta-hCG by bladder tumour cells.. J Mol Endocrinol..

[14] Butler S A, Iles R K (2004). The free monomeric beta subunit of human chorionic gonadotrophin (hCG) and the recently identified homodimeric beta-beta subunit (hCG beta beta) both have autocrine growth effects.. Tumour Biol..

[15] Butler S A, Ikram M S, Mathieu S, Iles R K (2000). The increase in bladder carcinoma cell population induced by free beta subunit of human chorionic gonadotrophin is a result of an anti-apoptosis effect and not cell proliferation.. Br J Cancer..

[16] Gillot D J, Iles R K, Chard T (1996). The Effects of beta-human chorionic gonadotrophin on the in vitro growth of bladder cancer cell lines.. Br J Cancer..

[17] Iles R K, Chard T (1989). Immunochemical analysis of the human chorionic gonadotrophin-like material secreted by 'normal' and neoplastic urothelial cells.. J Mol Endocrinol..

[18] Iles R K, Oliver R T, Kitau M, Walker C, Chard T (1987). In-vitro secretion of human chorionic gonadotrophin by bladder tumour cells.. Br J Cancer..

[19] Moutzouris G, Yannopoulos D, Barbatis C, Zaharof A, Theodorou C (1993). Is beta-human chorionic gonadotrophin production by transitional cell carcinoma of the bladder a marker of aggressive disease and resistance to radiotherapy?.. Br J Urol..

[20] Iles R K, Chard T (1989). Enhancement of ectopic beta-human chorionic gonadotrophin expression by interferon-alpha.. J Endocrinol..

[21] Hotakainen K, Lintula S, Jarvinen R, Paju A, Stenman J, Rintala E, Stenman U H (2007). Overexpression of human chorionic gonadotropin beta genes 3, 5 and 8 in tumor tissue and urinary cells of bladder cancer patiebts.. Tumour Biol..

[22] Iles R K, Jenkins B J, Oliver R T, Blandy j P, Chard T (1989). Beta human chorionic gonadotrophin in serum and urine A marker for metastatic urothelial cancer.. Br J Urol..

[23] Dobrowolski Z F, Byrska B, Dolezal M (1994). Prognostic value of beta human chorionic gonadotrophin in blood serum of patients with urinary bladder tumours.. Int Urol Nephrol..

[24] Smith D J, Evans H J, Newman J, Chapple C R (1994). Ectopic human chorionic gonadotrophin (HCG) production: is the detection by serum analysis of HCG of clinical relevance in transitional cell carcinoma of the bladder?.. Br J Urol..

[25] Mora J, Gascon N, Tabernero J M, Rodriguez-Espinosa J, Gonzalez-Sastre F (1996). Different hCG assays to measure ectopic hCG secretion in bladder carcinoma patients.. Br J Cancer..

[26] Iles R K, Persad R, Trivedi M, Sharma K B, Dickinson A, Smith P, Chard T (1996). Urinary concentration of human chorionic gonadotrophin and its fragments as a prognostic marker in bladder cancer.. Br J Urol..

[27] Venyo A K G, Herring D W, Shiel G (2001). Serum Human Chorionic Gonadotrophin in Human Urothelial Carcinoma.. African Journal of Urology..

[28] Lazar V, Diez S G, Laurent A, Giovangrandi Y, Radvany F, Chopin D, Bidart J M, Bellet D, Vidaud M (1995). Expression of human chorionic gonadotropin beta subunit genes in superficial and invasive bladder carcinomas.. Cancer Res..

[29] Geissler M, Wands G, Gesien A, de la Monte S, Bellet D, Wands J R (1997). Genetic immunization with the free human chorionic gonadotropin beta subunit elicits cytotoxic T lymphocyte responses and protects against tumor formation in mice.. Lab Invest..

[30] He L Z, Ramakrishna V, Connolly J E, Wang X T, Smith P A, Jones C L, Valkova-Valchanova M, Arunakumari A, Tremi J F, Goldstein J, Wallace P K, Keler T, Endres M J (2004). A novel human cancer vaccine elicits cellular responses to the tumor-associated antigen, human chorionic gonadotropin.. Clin Cancer Res..

